# Highly efficient lead removal from water by Nd_0.90_Ho_0.10_FeO_3_ nanoparticles and studying their optical and magnetic properties

**DOI:** 10.1038/s41598-023-43734-2

**Published:** 2023-10-03

**Authors:** M. M. Arman

**Affiliations:** Materials Science Lab (1), Physics Department, Faculty of Science, Cairo University, Giza, Egypt

**Keywords:** Materials science, Nanoscience and technology, Physics

## Abstract

Ho-doped NdFeO_3_ was synthesized using the citrate method. The X-ray diffraction (XRD) illustrated that Nd_0.90_Ho_0.10_FeO_3_ was crystalline at the nanoscale, with a crystallite size of 39.136 nm. The field emission scanning electron microscope (FESEM) illustrated the porous nature of Nd_0.90_Ho_0.10_FeO_3_, which increases the active sites to absorb the heavy metals on the sample surface. Energy-dispersive X-ray (EDX) data assures the prepared sample has the chemical formula Nd_0.90_Ho_0.10_FeO_3_. The magnetic properties of Nd_0.90_Ho_0.10_FeO_3_ were determined using the magnetization hysteresis loop and Faraday’s method. Many magnetic parameters of the sample have been discussed, such as the coercive field, the exchange bias (H_ex_), and the switching field distribution (SFD). Ho-doped NdFeO_3_ has an antiferromagnetic (AFM) character with an effective magnetic moment of 3.903 B.M. The UV–visible light absorbance of Nd_0.90_Ho_0.10_FeO_3_ is due to the transfer of electrons from the oxygen 2p state to the iron 3d state. Nd_0.90_Ho_0.10_FeO_3_ nanoparticles have an optical direct transition with an energy gap E_g_ = 1.106 eV. Ho-doped NdFeO_3_ can adsorb many heavy metals (Co^2+^, Ni^2+^, Pb^2+^, Cr^6+^, and Cd^2+^) from water. The removal efficiency is high for Pb^2+^ ions, which equals 72.39%. The Langmuir isotherm mode is the best-fit model for adsorbing the Pb^2+^ ions from water.

## Introduction

The orthoferrites are promising materials in many applications due to their chemical stability, magnetic, multiferroic, optical, and dielectric properties^1^. The general formula of orthoferrites is ABO_3_, where A is the rare earth elements, i.e., La^3+^, Nd^3+^, Sm^3+^, and Gd^3+^; B is the transition metal, i.e., Fe^3+^; and O^2-^ is the oxygen ion. Multiferroic materials such as BiFeO_3_, NdFeO_3_, SmFeO_3_, and LaFeO_3_ have ferroelectric and magnetic orders^2–5^. The applications of ABO_3_ are spintronics, data storage media, high-frequency devices, water purification, and photocatalysis^6–10^. The orthoferrites are characterized by low cost, chemical stability, easy fabrication, and many applications^11^.

NdFeO_3_ belongs to the ABO_3_ orthoferrite materials with the space group Pbnm. The Fe^3+^ ions form the FeO_6_ octahedron. The magnetic properties of NdFeO_3_ originate from the Dzyaloshinskii–Moriya exchange interaction of the antiparallel spins between Fe^3+^–O^2-^–Fe^3+^, Nd^3+^–O^2-^–Fe^3+^, and Nd^3+^–O^2-^–Nd^3+^^1,12^. The optical properties of NdFeO_3_ originate from the transition of electrons from 2p to 3d orbitals^13,14^. P. T. H. Duyen et al.^15^ studied the optical properties of Cd-doped NdFeO_3_, concluding that increasing the Cd concentration decreased the optical band gap. S. A. Mir et al.^16^ prepared the Ni-doped NdFeO_3_ and studied the dielectric properties of the samples.

Numerous photocatalysts, including CuS, TiO_2_, CuO, BaTiO_3_, ZnO, and others, are used today in dye degradation^17^. Their large bandgap energy, quick recombination of photoinduced charge carriers, and low visible light absorption, however, severely limit their practical applicability^18^. Therefore, it is essential to create photocatalysts with exceptional photocatalytic activity in the visible region and a small bandgap. Perovskite-oxide-based catalysts such as NdFeO_3_ have lately piqued the interest of researchers due to their good photocatalytic activity, tunable bandgap, high stability, and quick photoinduced electron/hole mobility^19–21^.

The metallic elements that are characterized by their high atomic weight, specific gravity, and toxicity are called heavy metals (HMs), such as lead (Pb^2+^), chromium (Cr^6+^), nickel (Ni^2+^), cadmium (Cd^2+^), and copper (Co^2+^). Cadmium, a heavy metal, damages the bones and kidneys^22^. Cr^6+^ causes hemorrhage, severe diarrhea, and cancer in the digestive tract^23^. Increasing the concentration of lead in drinking water causes kidney malfunction, brain tissue damage, and anemia^24^. There are many techniques used to remove heavy metals from water, such as the precipitation method^25^, flotation^26^, and membrane technologies^27^. The most effective method for removing HMs from water is the adsorption technique, which is characterized by its simplicity and no slugs.

The present paper describes the preparation of the Ho-doped NdFeO_3_ nanoparticles for the first time using a simple and inexpensive citrate combustion method. The sample was characterized by FESEM, EDX, and elementary mapping. The optical and magnetic properties of Nd_0.90_Ho_0.10_FeO_3_ were studied in detail. The removal of HMs (Co^2+^, Ni^2+^, Pb^2+^, Cr^6+^, and Cd^2+^) from water was studied. The Langmuir and Freundlich isotherm models were used to study the adsorption of Pb^2+^ from water on Nd_0.90_Ho_0.10_FeO_3_.

## Experimental work

### Materials

Neodymium nitrate, holmium nitrate, and iron nitrate were purchased from Sigma-Aldrich with a purity of 99.9%.

### Preparation of the Nd_0.90_Ho_0.10_FeO_3_ sample

**Figure 1:**
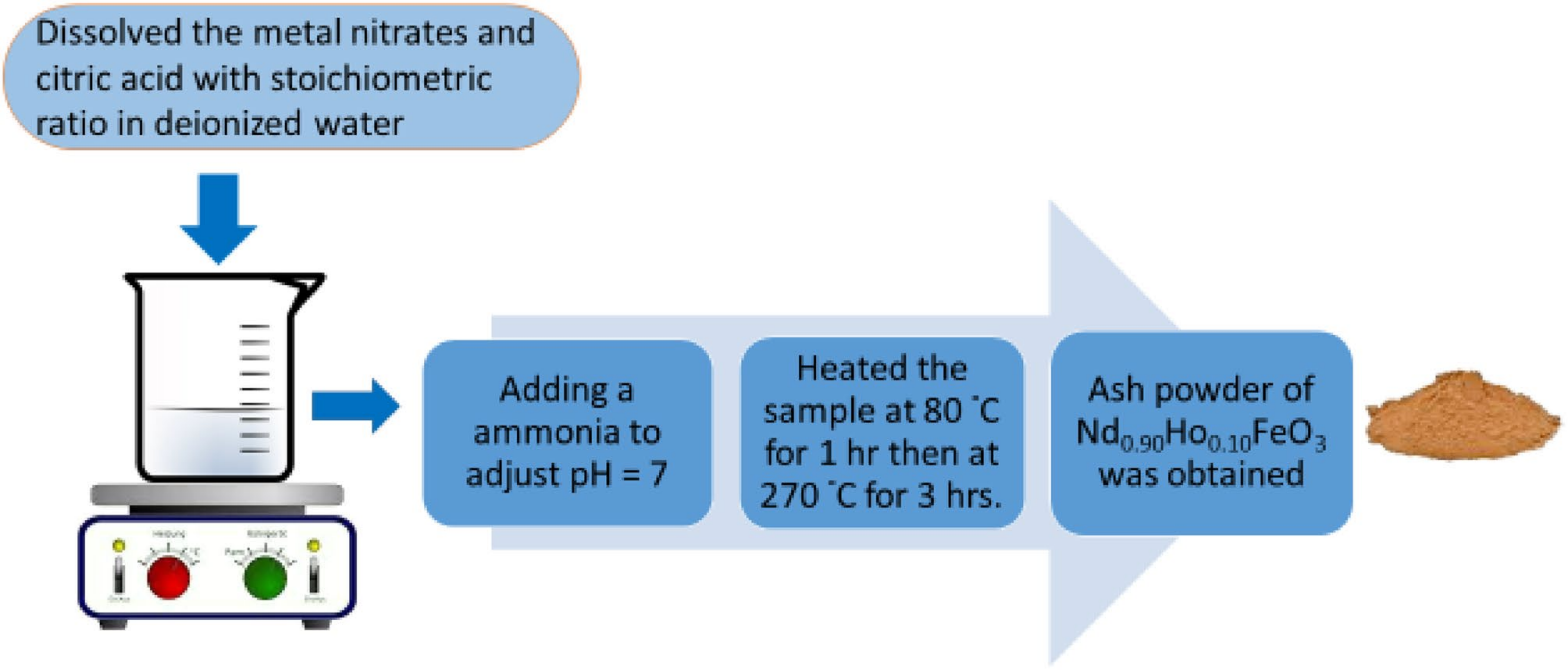
Flowchart for the preparation of Nd_0.90_Ho_0.10_FeO_3_.

The citrate combustion method is characterized by controlling the metal stoichiometry, high purity, low cost, crystallinity, effectiveness, and high yield. Figure 1 shows the flowchart of the synthesis of Nd_0.90_Ho_0.10_FeO_3_ using the citrate combustion method. The (0.9 M) Nd nitrate, (0.1 M) Ho nitrates, (1 M) Fe nitrates, and (2 M) citric acid were dissolved in distilled water. The ammonia solution was used to adjust the pH to 7. The solution was stirred and heated on a magnetic stirrer at 80 °C for one hour, then heated at 270 °C for 3 h until the evolution of fumes stopped. The as-prepared sample was ground using a mortar for one hour. The obtained powder was characterized by XRD to study the crystallinity of the Ho-doped NdFeO_3_.

### Nd_0.90_Ho_0.10_FeO_3_ characterizations and measurements

The crystal structure of Nd_0.90_Ho_0.10_FeO_3_ was studied using XRD (Bruker Advance D8 diffractometer, λ = 1.5406Å) with 2θ in the range of 20°–80°. The XRD data was indexed with the International Centre for Diffraction Data (ICDD) card number 01-089-6644. The morphology of Nd_0.90_Ho_0.10_FeO_3_ was studied using FESEM (model Quanta 250) with EDX and elemental mapping. The magnetic properties of Nd_0.90_Ho_0.10_FeO_3_ were studied by two techniques. The first is measuring the magnetization of the sample using the vibrating sample magnetometer (VSM; 9600-1 LDJ, USA), which uses a magnetic field up to 20 kOe at a temperature of 300 K. The second technique is Faraday’s method, in which a small amount of Nd_0.90_Ho_0.10_FeO_3_ was placed in a glass that was placed at the field gradient to measure the DC magnetic susceptibility with temperature^28^. The optical properties of Nd_0.90_Ho_0.10_FeO_3_ were studied using a UV–visible spectrophotometer (Jasco (V-630)).

### The heavy metals removal from water

The examination of the ability of Nd_0.90_Ho_0.10_FeO_3_ to remove heavy metals such as Co^2+^, Ni^2+^, Pb^2+^, Cr^6+^, and Cd^2+^ from water. The removal efficiency is represented by the following steps:

1. The standard solutions (50 ppm) of heavy metals were prepared.
2. 10 mL of the standard solutions were added to a beaker with 0.02 g of the sample.
3. The pH value of the solution was adjusted using the ammonia solution or diluted nitric acid.
4. The beakers were stirred on the electric shaker for 1 h at 170 rpm.
5. Take 8 mL of the solutions using a syringe filter.
6. Inductively coupled plasma spectrometry (ICP, Prodigy 7) was used to determine the concentration of the heavy metals.

## Results and discussion

**Figure 2:**
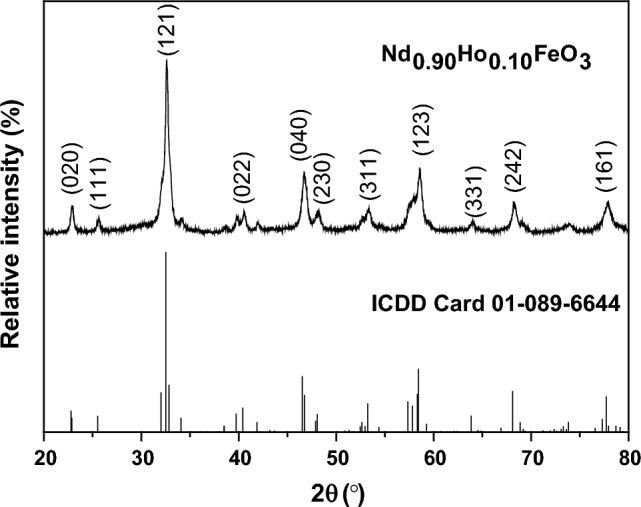
XRD of Nd_0.90_Ho_0.10_FeO_3_, ICDD card number 01-089-6644.

Figure 2 illustrates the XRD of the Nd_0.90_Ho_0.10_FeO_3_ nanoparticles. The most intense peak was observed at 2θ = 32.593º which characterized the (121) plane. The sample has a single phase orthorhombic structure. The average lattice parameters were estimated from all peaks using the following equation^29^:

1 $$ \frac{1}{{d^{2} }} = \frac{{h^{2} }}{{a^{2} }} + \frac{{k^{2} }}{{b^{2} }} + \frac{{l^{2} }}{{c^{2} }} $$

The average lattice parameters are reported in Table 1. The crystallite unit cell volume was calculated according to Eq. (2).

**Table 1:** The lattice parameters, the theoretical density (D_x_), the crystallite size (L), and the tolerance factor for Nd_0.90_Ho_0.10_FeO_3_.

Sample	a (Å) (0.0001)	B (Å) (0.0001)	C (Å) (0.0001)	V (Å^3^) (0.1)	D_x_ (g/cm^3^) (0.0002)	L (nm) (0.5)	t (0.0002)
Nd_0.90_Ho_0.10_FeO_3_	5.5696	7.7527	5.4561	235.6	7.0519	39	0.8875

2 $$V=abc$$

The average crystallite size (L) of the sample was estimated from all peaks using the Scherer formula, which is represented by the following equation^30^:

3 $$L=\frac{0.94\lambda }{\beta \mathit{cos}\theta }$$

where λ denotes the X-ray wavelength, β is the full width at half maximum, and θ is the Bragg angle. The value of L is 39.136 nm, which indicates the sample was prepared at the nanoscale.

The tolerance factor relates to the symmetry of the crystal structure and was calculated using Eq. (4).

4 $$\mathrm{t}=\frac{{\mathbf{r}}_{\mathbf{A}}+{\mathbf{r}}_{\mathbf{O}}}{\sqrt{2}({\mathbf{r}}_{\mathbf{F}\mathbf{e}}+{\mathbf{r}}_{\mathbf{O}})}$$

where r_A_, r_Fe_, and r_O_ are the ionic radii of the A, Fe, and oxygen ions, respectively. The value of r_A_ was calculated from Eq. (5).

5 $$ {\text{r}}_{{\text{A}}} = \, 0.{9}0\;r_{Nd}^{3 + } + 0.{1}0\; r_{Ho}^{2 + } $$

The value of t is one for the ideal cubic structure, while t decreases to one for the orthorhombic structure, where the crystallite size distortion increases. For the investigated sample, the value of t is 0.8875, which indicates the orthorhombic structure of the sample. The theoretical density (D_x_) was calculated according to Eq. (6) and reported in Table 1.

6 $${D}_{x}=\frac{ZM}{NV}$$

where Z is the number of molecules in a unit cell (Z = 4), M refers to the molecular weight of the sample, and N is Avogadro's number. The substitution of Ho^3+^ ions at the expense of the Nd^3+^ ion led to an increase in the relative density of NdFeO_3_. According to M.M. Arman^10^, the relative density of NdFeO_3_ is 6.33 g/cm^3^ and its unit cell volume is 236.9 (Å)^3^, while Nd_0.90_Ho_0.10_FeO_3_ has a relative density of 7.05 g/cm^3^ and its unit cell volume is 235.6 (Å)^3^. This is due to the ionic radius of Ho^3+^ ions (1.073 Å) being less than that of Nd^3+^ ions (1.163 Å)^31^. 

**Figure 3:**
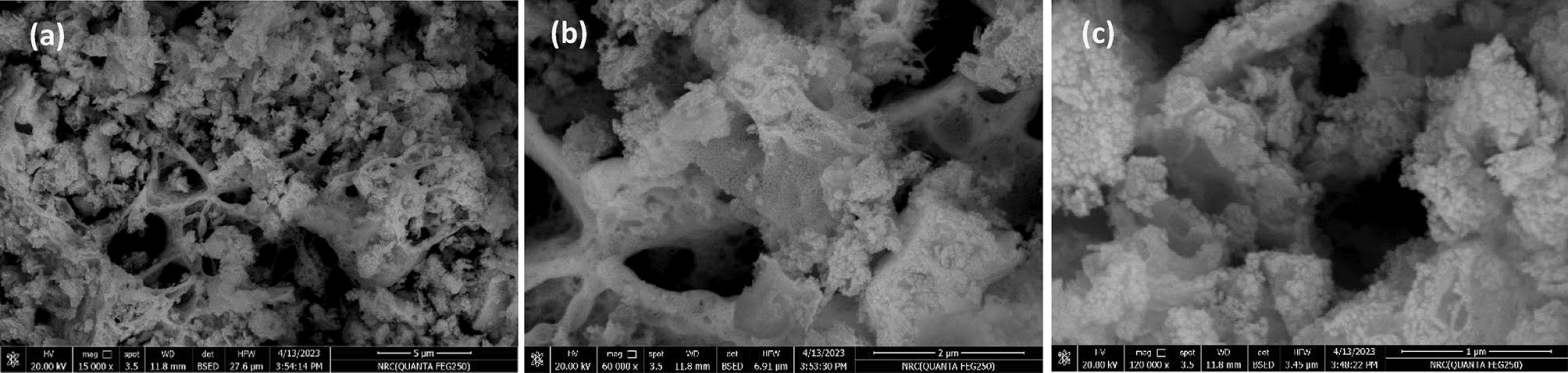
FESEM images of Nd_0.90_Ho_0.10_FeO_3_ with different magnifications.

Figure 3 illustrates the FESEM image of the Nd_0.90_Ho_0.10_FeO_3_ nanoparticles. The agglomerated particles are a result of the synthesis procedure. The sample has a porous nature, which increases the surface area of Ho-doped NdFeO_3_. The presence of a lot of active sites of the Nd_0.90_Ho_0.10_FeO_3_ increases the adsorption of HMs on the surface of the Nd_0.90_Ho_0.10_FeO_3_ sample^3^.

**Figure 4:**
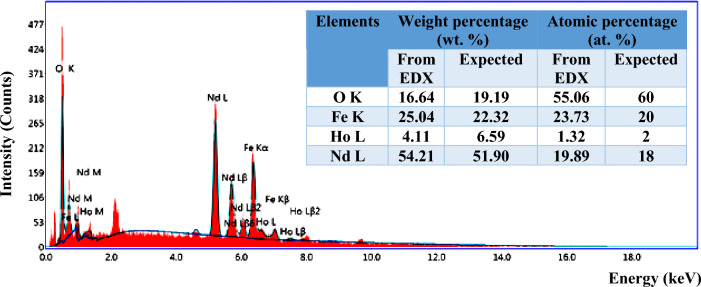
EDX for the Nd_0.90_Ho_0.10_FeO_3_ nanoparticles. The inset table shows the at% and wt% of the Ho, O, Fe, and Nd elements.

Figure 4 shows the EDX of Ho-doped NdFeO_3_, which assures the presence of the elements Fe, Ho, Nd, and O in the Nd_0.90_Ho_0.10_FeO_3_. The table inset in Fig. 4 shows the atomic percentage (at%) and weight percentage (wt%) of the elements, which were calculated theoretically from the sample formula and experimentally from the EDX data. The values of at% and wt% of the theoretical and experimental are close to each other, which indicates that the sample was prepared in the same chemical formula, Nd_0.90_Ho_0.10_FeO_3_. The peak observed at 2.11 eV is due to the gold coating of the sample before scanning. In Fig. 4, the carbon ions were observed at 0.27 eV due to the carbon tap where the sample was put inside the FESEM. The slight difference in wt% and at% between experimental and theoretical values is caused by the oxygen deficiency.

**Figure 5:**
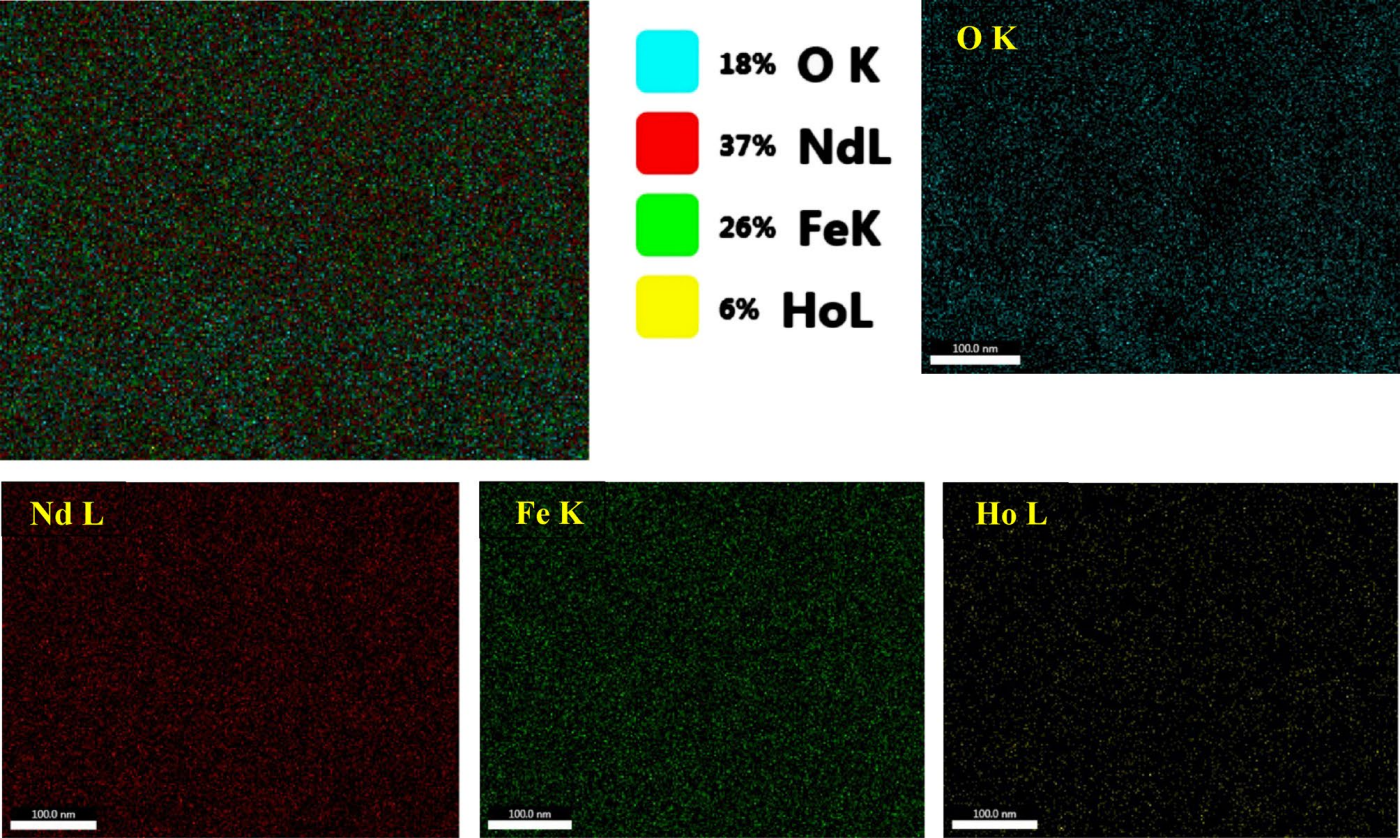
The elemental mapping of Nd_0.90_Ho_0.10_FeO_3_ nanoparticles.

Figure 5 shows the mapping of the elements in Ho-doped NdFeO_3_. Figure 5.a illustrates the homogeneous distribution of the elements in the sample. Figure 5b–e shows the distribution of each element in the sample by distinguished color. The wt% of the elements appearing in the elemental mapping was different from that obtained from EDX analysis due to the maps having been measured for too short a time.

The magnetic properties of Nd_0.90_Ho_0.10_FeO_3_ were studied via the M–H hysteresis loop and Faraday’s method. The magnetic behavior of Nd_0.90_Ho_0.10_FeO_3_ originates from the magnetic coupling between the magnetic ions such as Fe^3+^, Nd^3+^, and Ho^3+^ ions.

**Figure 6:**
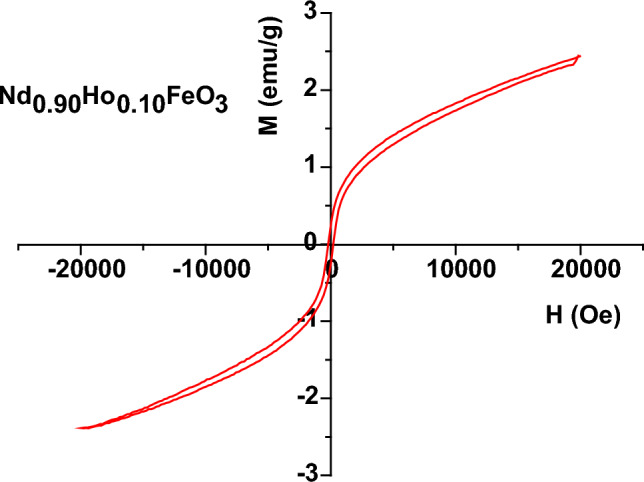
Magnetization (M–H) curve of Nd_0.90_Ho_0.10_FeO_3_ nanoparticles.

Figure 6 illustrates the magnetization hysteresis loop of Nd_0.90_Ho_0.10_FeO_3_, which has AFM behavior with weak ferromagnetic (FM) components^32^. The values of the saturation magnetization (M_s_) and coercive field (H_c_) are reported in Table 2. The value of the squareness ratio (SQR) of the sample was calculated from Eq. (7).

**Table 2:** The values of remanence magnetization (M_r_), the coercive field (H_c_), the exchange bias (H_ex_), the anisotropy constant (K), the switching field distribution (SFD), the rectangularity of the H-M loop (H_a_), and the squareness ratio for the sample.

Sample	M_s_ (emu/g)	M_r_ (emu/g)	H_c_ (Oe)	SQR = M_r_/M_s_	H_ex_ (Oe)	K (erg/g)	SDF	H_m_ (Oe)	H_a_ (Oe)
Nd_0.90_Ho_0.10_FeO_3_	2.42	0.25	200	0.103	− 10.86	504.17	2.33	255	417

7 $$SQR= \frac{{M}_{r}}{{M}_{s}}$$

The value of SQR was reported in Table 2 and indicates that the type of magnetic interaction is magneto-static interactions^33^. The exchange bias (H_ex_) of the sample was calculated from the Eq. (8).

8 $${H}_{ex}= \frac{- [{H}_{left}+ {H}_{right} ]}{2}$$

where H_left_ and H_right_ are the intercepts of the MH curve with the negative and positive x-axis, respectively. The presence of a shift in the MH loop around the origin originated from the presence of AFM ordering with (FM) spins in the sample.

The anisotropy constant (K) of Nd_0.90_Ho_0.10_FeO_3_ was calculated from Eq. (9)^34^:

9 $$K=\frac{{H}_{C}\times {M}_{S}}{0.96}$$

where H_c_ is the coercive field of the sample. The value of K was reported in Table 2.

**Figure 7:**
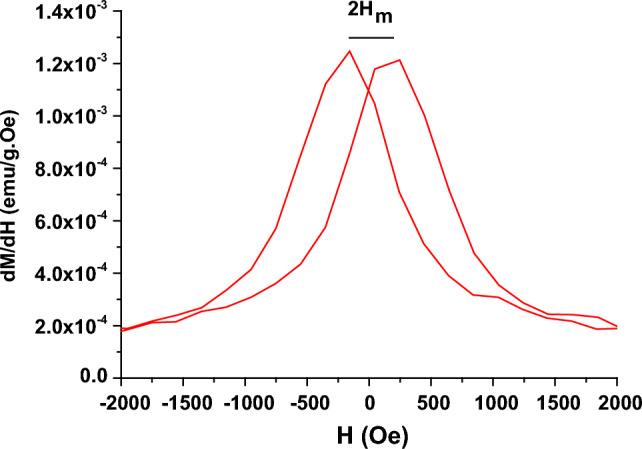
The relation between dM/dH and the magnetic field for Nd_0.90_Ho_0.10_FeO_3_ nanoparticles.

Figure 7 shows the dependence of dM/dH on the magnetic field for Nd_0.90_Ho_0.10_FeO_3_ nanoparticles. The switching field distribution (SFD) and the rectangularity of the H-M loop (H_a_) of Nd_0.90_Ho_0.10_FeO_3_ were calculated using the following equations^9^.

10 $$SFD= \frac{\Delta H}{{H}_{C}}$$

11 $${H}_{a}= \frac{2K}{{M}_{s}}$$

where ∆H represents the half width at the half maximum of the dM/dH peak. Table 2 contains the values of SFD and H_a_.

Many researchers have studied the preparation and properties of NdFeO_3_. M.M. Arman^10^ prepared the NdFeO_3_ nanoparticles using the citrate combustion method. The values of M_s_ and M_r_ of NdFeO_3_ are 1.05 emu/g and 0.11 emu/g, respectively. T. Shalini et al.^35^ studied the structure and magnetic behavior of NdFeO_3_, which has M_s_ and M_r_ equal to 0.521 emu/g and 0.098 emu/g, respectively. In the presence of work, the substitution of Ho^3+^ ions instead of Nd^3+^ ions increased the magnetic properties of the NdFeO_3_ nanoparticles. Where the effective magnetic moment of Nd^3+^ is 1.14 μ_B_ while that of Ho^3+^ is 10.6 μ_B_^36^. The presence of Ho^3+^ in the NdFeO_3_ increases the magnetic interactions between the magnetic ions such as Ho^3+^–O^2-^–Ho^3+^, Ho^3+^–O^2-^–Nd^3+^, and Ho^3+^–O^2—^Fe^3+^.‏

**Figure 8:**
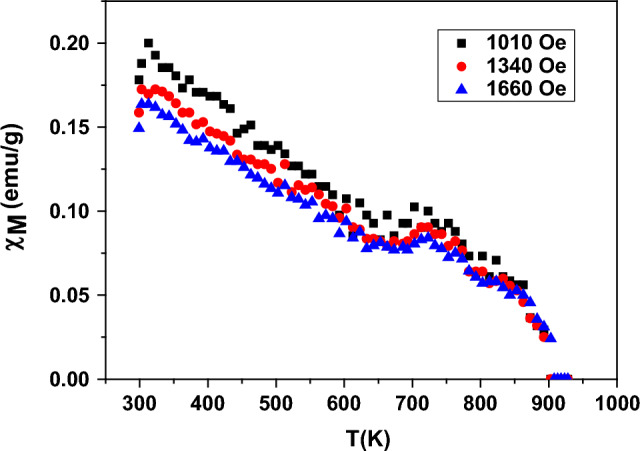
The dependence of χ_M_ on the temperature for Nd_0.90_Ho_0.10_FeO_3_.

Figure 8 shows the dependence of the molar magnetic susceptibility (χ_M_) on T(K). The behavior of χ_M_ with temperature assures that the sample has AFM behavior. χ_M_ decreases with raising the temperature up to the Neel temperature (T_N_), after which Nd_0.90_Ho_0.10_FeO_3_ has a paramagnetic behavior. The AFM properties originate from the magnetic interaction between the Fe^3+^ ions. The relation between χ_M_ and the applied field is inversely proportional according to the following equation:

12 $${\chi }_{M}= \frac{M}{H}$$

**Figure 9:**
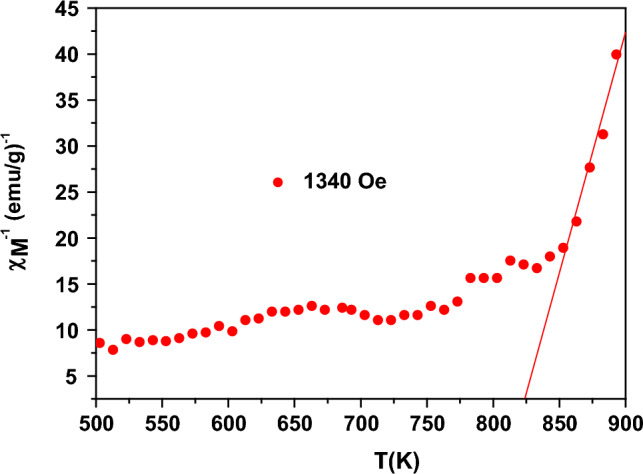
The relation of χ_M_^-1^ to the temperature at 1340 Oe.

The relation between χ_M_^-1^ and the temperature is shown in Fig. 9, which applies the Curie–Weiss law. From the slope of the paramagnetic region in Fig. 9, the values of the effective magnetic moment (μ_eff_), the Curie–Weiss constant (θ), and the Curie constant (C) were determined. The value of the μ_eff_ was determined from the following equation:

13 $${\mu }_{eff}= 2.83\sqrt{C}$$

where C denotes the reciprocal of the slope in the paramagnetic part. The values of μ_eff_, θ, and C were reported in Table 3. The value of T_N_ was determined from the differentiation of χ_M_ (dχ_M_/dT) and listed in Table 3.

**Table 3:** Values of the Curie–Weiss constant (θ), the Curie constant (C), the effective magnetic moment (μ_eff_), and the Neel temperature (T_N_) of Nd_0.90_Ho_0.10_FeO_3_ nanoparticles.

Sample	Field (Oe)	C (emu/g.mol) K	θ (K)	μeff (B.M.)	TN (K)
Nd_0.90_Ho_0.10_FeO_3_	1340	1.875	824.84	3.903	895.84

**Figure 10:**
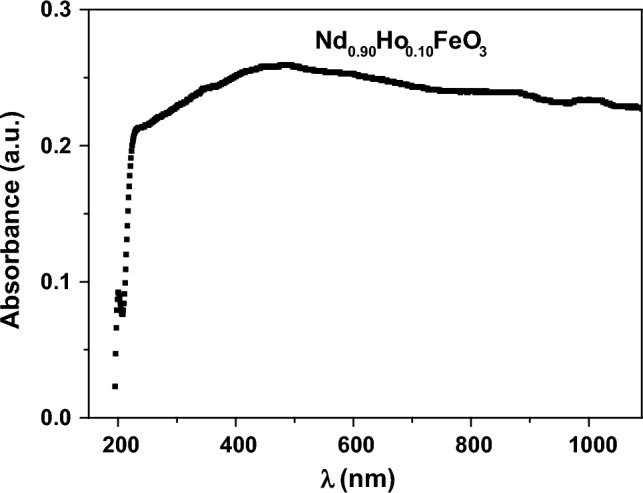
The relation between the absorbance and the wavelength of the photons for Nd_0.90_Ho_0.10_FeO_3_.

Figure 10 shows the dependence of the absorbance of the UV–visible light by the sample on the wavelength of the incident photons. In the low wavelength region (λ ≤ 460 nm), the absorbance increases rapidly with λ due to increasing the energy of the photons, which allows the electrons to be transferred from the 2p orbital oxygen valance band (V.B.) to the 3d orbital iron conduction band (C.B.). In the high wavelength region (λ > 460 nm), the energy of the photons is low and can’t transfer the electrons from V.B. to C.B.

**Figure 11:**
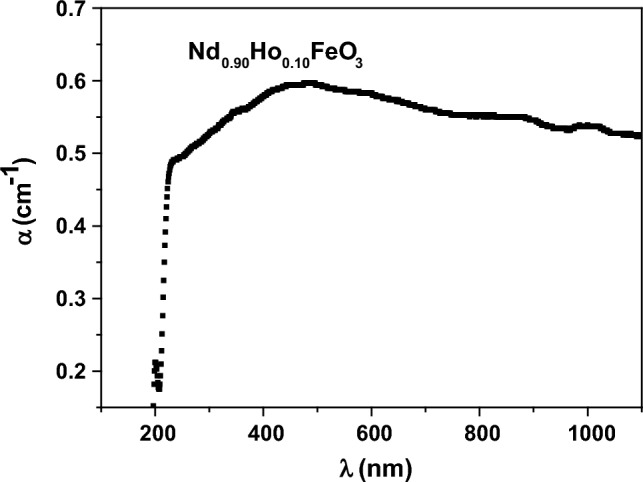
The dependence of α and the λ of the photons.

The optical absorption coefficient (α) is related to the quantity of UV–visible light absorption through the material. The value of α was determined using Eq. (14)^37^.

14 $$\alpha = \frac{\left(2.303\right) A}{l}$$

where A is the absorbance and l denotes the length of the spacemen. Figure 11 shows the relationship between α and the wavelength. The values of α increased rapidly with increasing λ up to 460 nm, then α decreased slowly with increasing λ. The increasing of α is due to increasing the absorption of photons at low wavelengths and higher frequencies, while the decreasing of α is due to decreasing the absorption of photons at high wavelengths and lower frequencies.

The optical extinction coefficient (k) denotes the losses of electromagnetic energy in Nd_0.90_Ho_0.10_FeO_3_ nanoparticles. k was calculated using the Eq. (15).

15 $$k= \frac{\alpha \lambda }{4\pi }$$

**Figure 12:**
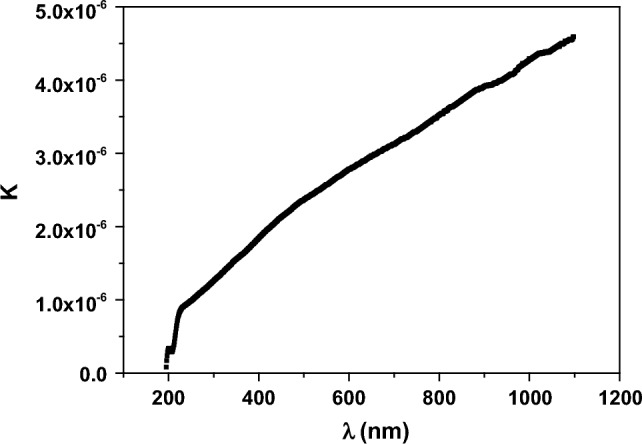
The relation between k and the wavelength for Nd_0.90_Ho_0.10_FeO_3_ nanoparticles.

Figure 12 studies the dependence of k on wavelength of the photons. The increasing of k with increasing λ of photons is due to the fact that at high λ, the energy of the photons is small and doesn’t absorb in the sample, increasing the energy losses, so k increases.

**Figure 13:**
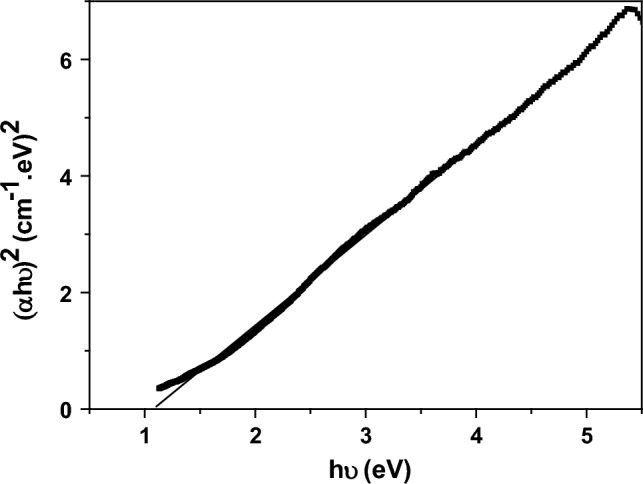
The Tauc plot for the optical direct transition of Nd_0.90_Ho_0.10_FeO_3_.

The Tauc plot was used to determine the optical band gap value (E_g_) and the type of optical transition of Nd_0.90_Ho_0.10_FeO_3_. The Tauc equation was represented by Eq. (16)^37^.

16 $$(\alpha hv{)}^{x}=A\left(hv-{E}_{g}\right)$$

where A and hν are the constant and photon energy, respectively. The (x) value was estimated to be the type of optical transition. For the direct transition, x equals 2, while for the indirect transition, x equals 1. The Tauc plot is represented in Fig. 13, which illustrates that the Nd_0.90_Ho_0.10_FeO_3_ sample has a direct transition with E_g_ = 1.106 eV. J. S. Prabagar et al.^21^ prepared NdFeO_3_ using the citrate sol–gel method with an optical bandgap of 2.48 eV and good photocatalytic activities. While introducing the Ho^3+^ ions in the NdFeO_3_ leads to a decrease in E_g_ to 1.106 eV due to introducing orbitals and states of Ho^3+^ ions in the NdFeO_3_ system. The author recommended using Nd_0.90_Ho_0.10_FeO_3_ as a photocatalyst for organic dye degradation in water.

**Figure 14:**
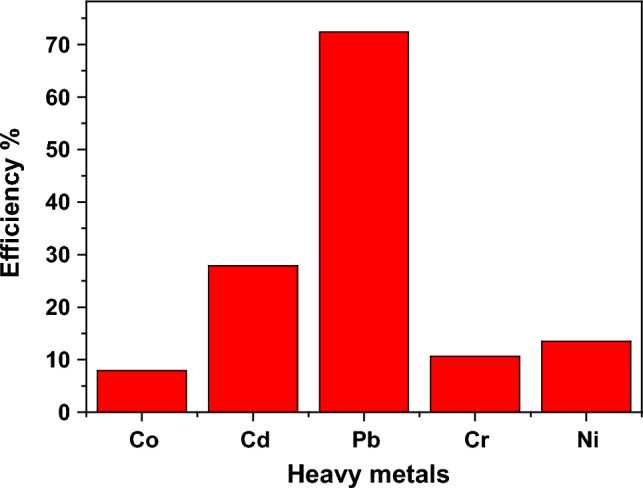
The removal effectiveness of Nd_0.90_Ho_0.10_FeO_3_ for the different heavy metals.

Figure 14 illustrates the dependence of the removal efficiency of the HMs at Ph = 7, which was calculated using Eq. (17).

17 $$\eta =\frac{{C}_{i}-{C}_{f}}{{C}_{i}} \times 100$$

where ${C}_{f}$ is the final concentration while and ${C}_{i}$ denotes the initial concentration of the HMs. The sample Nd_0.90_Ho_0.10_FeO_3_ has the ability to remove a lot of HMs from water, which indicates that it has a large surface area. The adsorption of the HMs from an aqueous solution depends on many parameters, such as temperature, pH value of the solution, contact time, ionic radii of the HMs, initial concentration of the adsorbent, and molecular weight of the HMs. The highest efficiency of HMs from the water was observed for Pb^2+^ ions with η = 72.39% due to the fact that Pb^2+^ ions have a higher molecular weight than the other HMs, leading to easier adsorption.

**Figure 15:**
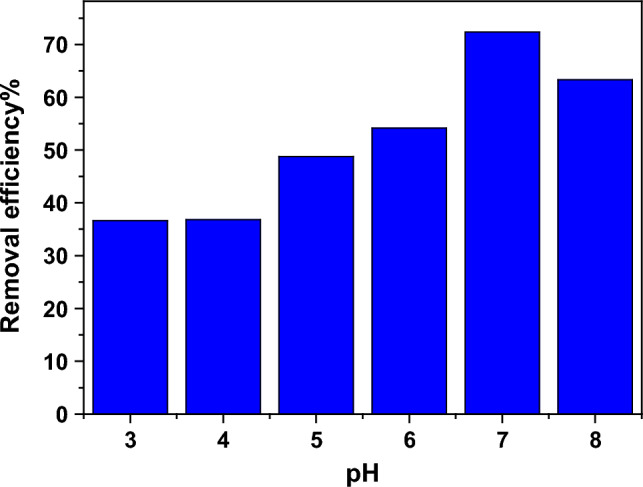
The dependence of the removal efficiency of Pb^2+^ ions on the pH value of the solution.

The effect of the pH value of the solution on the removal efficiency (η) of Pb^2+^ ions was studied and illustrated in Fig. 15. In the acidic region (pH ≤ 6), there are a lot of H^+^ ions in the solution, which participate with the Pb^2+^ ions in the adsorption on the active sites on the surface of Nd_0.90_Ho_0.10_FeO_3_, so η is low. The maximum adsorption of Pb^2+^ ions was observed at pH = 7, which indicates the optimum pH condition for removal of Pb^2+^ from aqueous solutions using Ho-doped NdFeO_3_. The FESEM images of the sample illustrate the porous nature of the surface of the sample, which increases the active sites that adsorb the HMs from the water. In the basic medium (pH = 8), the Pb^2+^ ions can be precipitated as lead hydroxide, which is not favorable.

The adsorption mechanism of the Pb^2+^ ions from the water was studied using the adsorption isotherm models. In the present work, the Langmuir and Freundlich isotherm models were used to study the adsorption of Pb^2+^ on the surface of the sample.

**Figure 16:**
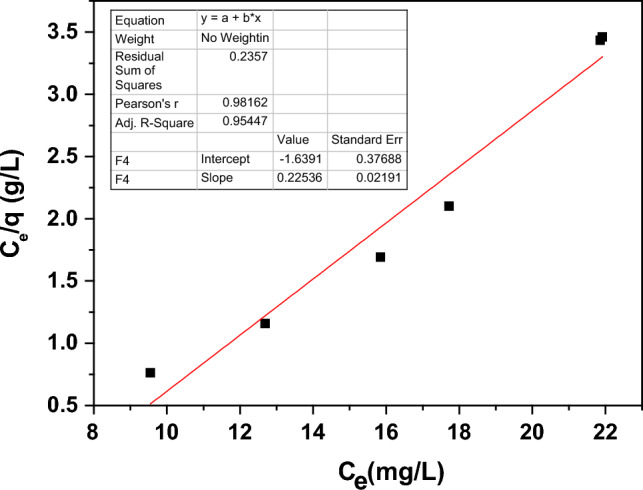
The Langmuir model fitting for Ho-doped NdFeO_3_.

The Langmuir isotherm represents the adsorption of the Pb^2+^ ions on the surface active sites as a single layer. The assumptions of the Langmuir isotherm are that the HMS is adsorbed on discrete surface active sites, each HM molecule adsorbs on one active site, the sample has a uniform adsorbing surface, and HM molecules don’t interact with each other^38^. The following equation describes the Langmuir isotherm mode.

18 $$\frac{{C}_{e}}{{q}_{e}}=\frac{1}{{q}_{m}{K}_{L} }+ \frac{{C}_{e}}{{q}_{m}}$$

where C_e_ is the equilibrium Pb^2+^ ion concentration, q_m_, and K_L_ denote the Langmuir constants. The equilibrium adsorption capacity (q_e_) was calculated using Eq. (19).

19 $${q}_{e}=\frac{\left({C}_{i}-{C}_{e}\right)V}{m}$$

where V and m are the volume of the Pb^2+^ solution and the adsorbent mass, respectively. Figure 16 shows the fitting of the experimental data with the Langmuir isotherm model.

**Figure 17:**
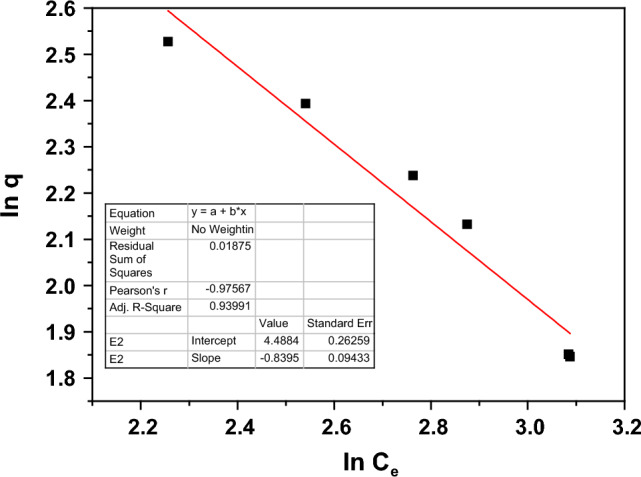
The Freundlich model fitting for Nd_0.90_Ho_0.10_FeO_3_.

The Freundlich isotherm describes the mass transportation of the Pb^2+^ ions from the aqueous solution to the active sites on the porous surface of Nd_0.90_Ho_0.10_FeO_3_. Equation (20) represents the Freundlich isotherm model.

20 $$\mathrm{Ln} {q}_{e}=\mathrm{Ln} {K}_{f}+ \frac{1}{n}\mathrm{Ln} {C}_{e}$$

where *K*_*f*_ denotes the Freundlich constant. Figure 17 shows the fitting of the experimental data with the Freundlich isotherm.

From the inset tables in Figs. 16 and 17, the values of R^2^ are 0.9545 and 0.9399 for the Langmuir and Freundlich isotherm modes, respectively. The Langmuir isotherm mode is the best-fit model for adsorbing the Pb^2+^ ions from water, and the HMs form monolayer adsorption.

## Conclusion

Nd_0.90_Ho_0.10_FeO_3_ was prepared in an orthorhombic structure with a crystallite size of 39.136 nm. FESEM illustrates the agglomerated grains due to the magnetic behavior of the sample. The EDX data shows that the elements Fe, Ho, Nd, and O are present in Ho-doped sample without any impurities. The antiferromagnetic properties of Nd_0.90_Ho_0.10_FeO_3_ originate from the magnetic coupling of Fe^3+^–O^2-^–Fe^3+^, Nd^3+^–O^2-^–Fe^3+^, and Nd^3+^–O^2-^–Nd^3+^. The values of M_s_, M_r_, H_c_, H_ex_, K, and SDF are 2.42 emu/g, 0.25 emu/g, 200 Oe, -10.86 Oe, 514.14 erg/g, and 2.33, respectively. The sample has a direct optical transition with E_g_ = 1.106 eV. Nd_0.90_Ho_0.10_FeO_3_ is a good absorber of UV–visible light and can be used for photocatalysis of organic dye degradation in water. The Nd_0.90_Ho_0.10_FeO_3_ nanoparticles have a high efficiency (72.39%) to remove the heavy metal Pb^2+^ from water. The experimental data is more fitting for the Langmuir isotherm mode.

## Data Availability

The author declares that all the data supporting the findings of this study are available in the ICDD card number 01-089-6644.

## References

[1] Khaled MA, Ruvalcaba J, Córdova TF, El Marssi M, Bouyanfif H (2022). Spin-lattice coupling in an epitaxial NdFeO_3_ thin film. Mater. Lett..

[2] Kumar S (2021). Compositional-driven multiferroic and magnetoelectric properties of NdFeO_3_-PbTiO_3_ solid solutions. J. Asian Ceram. Soc..

[3] Arman MM (2023). Novel multiferroic nanoparticles Sm_1-x_Ho_x_FeO_3_ as a heavy metal Cr^6+^ ion removal from water. Appl. Phys. A.

[4] Ahmed MA, Selim MS, Arman MM (2011). Novel multiferroic La_0.95_Sb_0.05_FeO_3_ orthoferrite. Mater. Chem. Phys..

[5] Jia DC, Xu JH, Ke H, Wang W, Zhou Y (2009). Structure and multiferroic properties of BiFeO_3_ powders. J. Eur. Ceram. Soc..

[6] Cheng Y, Peng B, Hu Z, Zhou Z, Liu M (2018). Recent development and status of magnetoelectric materials and devices. Phys. Lett. A.

[7] Vopson MM (2015). Fundamentals of multiferroic materials and their possible applications. Crit. Rev. Solid State Mater. Sci..

[8] Yadav SK, Hemalatha J (2022). Electrospinning and characterization of magnetoelectric NdFeO_3_–PbZr_0.52_Ti_0.48_O_3_ core-shell nanofibers. Ceram. Int..

[9] Arman MM, Ramadan R (2023). Spherical SiO_2_ growth on LaFeO_3_ perovskite to create core–shell structures for Cd (II) adsorption on its surface. J. Mater. Sci. Mater. Electron..

[10] Arman MM (2023). The effect of the rare earth A-site cation on the structure, morphology, physical properties, and application of perovskite AFeO_3_. Mater. Chem. Phys..

[11] Bammannavar BK, Naik LR (2012). Study of magnetic properties and magnetoelectric effect in (x)Ni_0.5_Zn_0.5_Fe_2_O_4_+ (1–x) PZT composites. J. Magn. Magn. Mater..

[12] Yuan SJ (2013). Spin switching and magnetization reversal in single-crystal NdFeO_3_. Phys. Rev. B.

[13] Bharadwaj PSJ, Kundu S, Kollipara VS, Varma KB (2019). Structural, optical and magnetic properties of Sm^3+^ doped yttrium orthoferrite (YFeO_3_) obtained by sol–gel synthesis route. J. Phys. Condens. Matter..

[14] Wang M, Wang T (2019). Structural, magnetic and optical properties of Gd and Co co-doped YFeO_3_ nanopowders. Materials.

[15] Duyen PTH, Diem CH, Tien NA (2022). Cd-doped NdFeO_3_ nanoparticles: Synthesis and optical properties study. J. Mater. Sci. Mater. Electron..

[16] Mir SA, Ikram M, Asokan K (2014). Structural, optical and dielectric properties of Ni substituted NdFeO_3_. Optik.

[17] Shivaraju HP, Yashas SR, Harini R (2020). Application of Mg-doped TiO_2_ coated buoyant clay hollow-spheres for photodegradation of organic pollutants in wastewater. Mater. Today Proc..

[18] Shanmugam V, Jeyaperumal KS, Mariappan P, Muppudathi AL (2020). Fabrication of novel gC_3_N_4_ based MoS_2_ and Bi_2_O_3_ nanorod embedded ternary nanocomposites for superior photocatalytic performance and destruction of bacteria. New J. Chem..

[19] Abdi M, Mahdikhah V, Sheibani S (2020). Visible light photocatalytic performance of La-Fe co-doped SrTiO_3_ perovskite powder. Opt. Mater..

[20] Ismael M, Wark M (2019). Perovskite-type LaFeO_3_: Photoelectrochemical properties and photocatalytic degradation of organic pollutants under visible light irradiation. Catalysts.

[21] Prabagar JS, Tenzin T, Yadav S, Kumar KMA, Shivaraju HP (2023). Facile synthesis of NdFeO_3_ perovskite for photocatalytic degradation of organic dye and antibiotic. Mater. Today Proc..

[22] Godt J (2006). The toxicity of cadmium and resulting hazards for human health. J. Occup. Med. Toxicol..

[23] Alemu A, Lemma B, Gabbiye N (2019). Adsorption of chromium (III) from aqueous solution using vesicular basalt rock. Cogent Environ. Sci..

[24] Brooks RM, Bahadory M, Tovia F, Rostami H (2010). Removal of lead from contaminated water. Int. J. Soil Sediment Water.

[25] Mbamba CK, Batstone DJ, Flores-Alsina X, Tait S (2015). A generalised chemical precipitation modelling approach in wastewater treatment applied to calcite. Water Res..

[26] Da Silva SS, Chiavone-Filho O, de Barros Neto EL, Foletto EL (2015). Oil removal from produced water by conjugation of flotation and photo-Fenton processes. J. Environ. Manag..

[27] Neoh CH, Noor ZZ, Mutamim NSA, Lim CK (2016). Green technology in wastewater treatment technologies: Integration of membrane bioreactor with various wastewater treatment systems. Chem. Eng. J..

[28] Arman MM (2022). Synthesis, characterization, magnetic properties, and applications of La_0.85_Ce_0.15_FeO_3_ perovskite in heavy metal Pb^2+^ removal. J. Supercond. Nov. Magn..

[29] Arman MM (2023). Preparation, characterization and magnetic properties of Sm_0.95_Ho_0.05_FeO_3_ nanoparticles and their application in the purification of water. Appl. Phys. A.

[30] Arman MM, Gamal AAR (2023). Role of Gd^3+^ and Ho^3+^ doping on the structure, physical properties and applications of ZnO. Appl. Phys. A.

[31] Shannon RD (1976). Revised effective ionic radii and systematic studies of interatomic distances in halides and chalcogenides. Acta Crystallogr. Sect. A Cryst. Phys. Diffr. Theor. Gen. Crystallogr..

[32] Zhou JS, Marshall LG, Li ZY, Li X, He JM (2020). Weak ferromagnetism in perovskite oxides. Phys. Rev. B.

[33] Jiles DC (2003). Recent advances and future directions in magnetic materials. Acta Mater..

[34] Ateia EE, Ateia MA, Arman MM (2022). Assessing of channel structure and magnetic properties on heavy metal ions removal from water. J. Mater. Sci. Mater. Electron..

[35] Shalini T, Vijayakumar P, Kumar J (2020). Studies on structural and magnetic properties of NdFeO_3_ single crystals grown by optical floating zone technique. Bull. Mater. Sci..

[36] Morosan E (2008). Structure and magnetic properties of the Ho_2_Ge_2_O_7_ pyrogermanate. Phys. Rev. B.

[37] Arman MM, Ahmed MK, El-Masry MM (2023). Cellulose Acetate polymer spectroscopic study comprised LaFeO_3_ perovskite and graphene as a UV-to-visible light converter used in several applications. J. Mol. Struct..

[38] Goodfellow, H. D. & Wang, Y. (2^nd^ Eds.). *Industrial Ventilation Design Guidebook: Volume 2: Engineering Design and Applications* (Academic press, 2021).‏

